# A Severe Lack of Evidence Limits Effective Conservation of the World's Primates

**DOI:** 10.1093/biosci/biaa082

**Published:** 2020-08-26

**Authors:** Jessica Junker, Silviu O Petrovan, Victor Arroyo-RodrÍguez, Ramesh Boonratana, Dirck Byler, Colin A Chapman, Dilip Chetry, Susan M Cheyne, Fanny M Cornejo, Liliana CortÉs-Ortiz, Guy Cowlishaw, Alec P Christie, Catherine Crockford, Stella De La Torre, Fabiano R De Melo, P Fan, Cyril C Grueter, Diana C GuzmÁn-Caro, Eckhard W Heymann, Ilka Herbinger, Minh D Hoang, Robert H Horwich, Tatyana Humle, Rachel A Ikemeh, Inaoyom S Imong, Leandro Jerusalinsky, Steig E Johnson, Peter M Kappeler, Maria CecÍlia M Kierulff, Inza KonÉ, Rebecca Kormos, Khac Q Le, Baoguo Li, Andrew J Marshall, Erik Meijaard, Russel A Mittermeier, Yasuyuki Muroyama, Eleonora Neugebauer, Lisa Orth, Erwin Palacios, Sarah K Papworth, Andrew J Plumptre, Ben M Rawson, Johannes Refisch, Jonah Ratsimbazafy, Christian Roos, Joanna M Setchell, Rebecca K Smith, Tene Sop, Christoph Schwitzer, Kathy Slater, Shirley C Strum, William J Sutherland, MaurÍcio Talebi, Janette Wallis, Serge Wich, Elizabeth A Williamson, Roman M Wittig, Hjalmar S KÜhl

**Affiliations:** German Centre for Integrative Biodiversity Research, Leipzig, Germany and with the Max Planck Institute for Evolutionary Anthropology, formerly the Department of Primatology, in Leipzig, Germany; Conservation Science Group, Department of Zoology, University of Cambridge, Cambridge, United Kingdom; Instituto de Investigaciones en Ecosistemas y Sustentabilidad, Universidad Nacional Autónoma de México, Morelia, Mexico; Mahidol University International College, Salaya, Thailand; Global Wildlife Conservation, Austin, Texas; Department of Anthropology, McGill University, Montreal, Quebec, Canada; with the School of Life Sciences, University of KwaZulu-Natal, Scottsville, Pietermaritzburg, South Africa; and with the Shaanxi Key Laboratory for Animal Conservation, at Northwest University, in Xi'an, China; Gibbon Conservation Centre, Assam, India; Borneo Nature Foundation, Palangka Raya, Central Kalimantan, Indonesia, and with the Department of Social Sciences, Oxford Brookes University, Oxford, United Kingdom; Stony Brook University, Stony Brook, New York; Department of Ecology and Evolutionary Biology, University of Michigan, Ann Arbor, Michigan; Institute of Zoology, Zoological Society of London, London, in the United Kingdom; Conservation Science Group, Department of Zoology, University of Cambridge, Cambridge, United Kingdom; Tai Chimpanzee Project, Centre Suisse des Recherche Scientifique, Abidjan, Cote d'Ivoire; Universidad San Francisco de Quito's Colegio de Ciencias Biológicas y Ambientales in Quito, Ecuador; Department of Engenharia Florestal, Federal University of Viçosa, Viçosa, Brazil; School of Life Sciences, Sun Yat-Sen University, Guangzhou, China; School of Human Sciences and with the School of Biological Sciences, University of Western Australia, Crawley, Western Australia, Australia; Asociación Primatológica Colombiana, Bogotá, Colombia; Deutsches Primatenzentrum, Leibniz-Institut für Primatenforschung, Göttingen, Germany; World Wide Fund for Wildlife Germany, Berlin, Germany; Southern Institute of Ecology, Hochiminh City, Vietnam; Community Baboon Sanctuary, Belize; Durrell Institute of Conservation and Ecology, School of Anthropology and Conservation, University of Kent, Kent, United Kingdom; SW/Niger Delta Forest Project, part of the Foundation for Sustainability of Ecosystem, Wildlife, and Climate, Abuja, Nigeria; Wildlife Conservation Society Nigeria, Calabar, Nigeria; Centro Nacional de Pesquisa e Conservação de Primatas Brasileiros, in the Instituto Chico Mendes de Conservação da Biodiversidade. In João Pessoa, Brazil; Department of Anthropology and Archaeology, University of Calgary, Calgary, Alberta, Canada; Deutsches Primatenzentrum, Leibniz-Institut für Primatenforschung, Göttingen, Germany, and with the Department of Sociobiology/Anthropology, Faculty of Biology and Psychology, at Georg-August Universität, in Göttingen, Germany; Instituto Nacional da Mata Atlântica, in Espírito Santo, Brazil, and with the Instituto Pri-Matas, Minas Gerais, Brazil; Centre Suisse de Recherches Scientifiques, Abidjan, Côte d'Ivoire. Rebecca Kormos is affiliated with the Department of Integrative Biology, University of California, Berkeley; German Centre for Integrative Biodiversity Research, Leipzig, Germany and with the Max Planck Institute for Evolutionary Anthropology, formerly the Department of Primatology, in Leipzig, Germany; Freelance wildlife consultant, Hanoi, Vietnam; Shaanxi Key Laboratory for Animal Conservation, College of Life Sciences, Northwest University, Xi'an, China; Department of Anthropology and the Department of Ecology and Evolutionary Biology in the Program in the Environment and the School of Environment and Sustainability, Universit of Michigan in Ann Arbor, Michigan; Center of Excellence for Environmental Decisions, University of Queensland, Brisbane, Queensland, Australia, and with Borneo Futures, Bandar Seri Begawan, Brunei; Global Wildlife Conservation, Austin, Texas; Natural Science Laboratory, Faculty of Business Administration, Toyo University, Tokyo, Japan; Universität Leipzig, Dekanat der Fakultät für Lebenswissenschaften, Leipzig, Germany; Independent researcher, Leipzig, Germany; Conservación Internacional, Colombia, Bogotá; Royal Holloway University of London, Egham, United Kingdom; Department of Anthropology and the Department of Ecology and Evolutionary Biology in the Program in the Environment and the School of Environment and Sustainability, Universit of Michigan in Ann Arbor, Michigan; BirdLife International, both in Cambridge, United Kingdom; World Wide Fund for Wildlife Vietnam, Hanoi, Vietnam; Great Apes Survival Partnership, United Nations Environment Programme, Nairobi, Kenya; Groupe d’étude et de recherche sur les primates, Antananarivo, Madagascar; Deutsches Primatenzentrum, Leibniz-Institut für Primatenforschung, Göttingen, Germany; Department of Anthropology, Durham University, Durham, United Kingdom; Conservation Science Group, Department of Zoology, University of Cambridge, Cambridge, United Kingdom; Max Planck Institute for Evolutionary Anthropology, formerly the Department of Primatology, Leipzig, Germany; Bristol Zoological Society, Bristol Zoo Gardens, Bristol, United Kingdom; Operation Wallacea, Lincolnshire, United Kingdom; University of California San Diego, La Jolla, California, and with the Uaso Ngiro Baboon Project, Nairobi, Kenya; Conservation Science Group, Department of Zoology, University of Cambridge, Cambridge, United Kingdom; Departamento de Cíências Ambientais and the Programa Análise Ambiental Integrada, Universidade Federal de São Paulo, Sao Paulo, Brazil; Department of Environmental Studies, University of Oklahoma, Norman, Oklahoma; School of Natural Sciences and Psychology, Liverpool John Moores University, Liverpool, United Kingdom; Faculty of Natural Sciences, University of Stirling, Stirling, Scotland, United Kingdom; Tai Chimpanzee Project, Centre Suisse des Recherche Scientifique, Abidjan, Cote d'Ivoire; German Centre for Integrative Biodiversity Research, Leipzig, Germany and with the Max Planck Institute for Evolutionary Anthropology, formerly the Department of Primatology, in Leipzig, Germany

**Keywords:** conservation interventions, effectiveness, evidence based, IUCN SSC Primate Specialist Group

## Abstract

Threats to biodiversity are well documented. However, to effectively conserve species and their habitats, we need to know which conservation interventions do (or do not) work. Evidence-based conservation evaluates interventions within a scientific framework. The Conservation Evidence project has summarized thousands of studies testing conservation interventions and compiled these as synopses for various habitats and taxa. In the present article, we analyzed the interventions assessed in the primate synopsis and compared these with other taxa. We found that despite intensive efforts to study primates and the extensive threats they face, less than 1% of primate studies evaluated conservation effectiveness. The studies often lacked quantitative data, failed to undertake postimplementation monitoring of populations or individuals, or implemented several interventions at once. Furthermore, the studies were biased toward specific taxa, geographic regions, and interventions. We describe barriers for testing primate conservation interventions and propose actions to improve the conservation evidence base to protect this endangered and globally important taxon.

Threats to biodiversity and their consequences for the natural world are increasingly documented and understood. The International Union for Conservation of Nature (IUCN) regularly updates information on the status, threats, and population trends of over 100,000 species (IUCN 2020). This is an important step toward determining how to protect species effectively. However, monitoring trends alone is insufficient to prevent extinction, as was illustrated by the Christmas Island pipistrelle (*Pipistrellus murrayi*; Lindenmayer et al. 2013), the Yangtze River dolphin (*Lipotes vexillifer*; Turvey et al. 2007), and the scimitar-horned oryx (*Oryx dammah*; Gilbert and Woodfine 2004), although negative population trends can be reversed with effective conservation interventions, as was the case with golden lion tamarin (*Leontopithecus rosalia*; Kierulff et al. 2012), the black-footed ferret (*Mustela nigripes*; Grenier et al. 2007), and the mountain gorilla (*Gorilla beringei beringei*; Robbins et al. 2011). Where possible, management decisions should be informed by evidence of the effectiveness of conservation interventions for a given species. Analogous to evidence-based medicine, which relies on rigorous collation of data and evaluation of treatments to maximize success, evidence-based conservation evaluates interventions within a scientific framework (Sutherland et al. 2004).

Thousands of studies have evaluated a wide spectrum of conservation interventions, permitting a quantitative assessment of their effectiveness. There have been several attempts to compile the available evidence to empower policy and decision-makers by providing empirical support (or not) for specific interventions. For example, the Collaboration for Environmental Evidence (www.environmentalevidence.org) provides environmental evidence syntheses in the form of systematic reviews for specific conservation questions and well-defined interventions and outcomes or, alternatively, delivers systematic maps, which show the available evidence for broader questions involving many interventions and outcomes.

The Conservation Evidence project (www.conservationevidence.com) uses a slightly different approach. Together with international researchers and conservation practitioners from around the world, this initiative develops synopses of evidence, which provide a comprehensive database of studies that evaluate all conservation interventions that could be implemented for prespecified groups of species or habitats. For each intervention, relevant studies are grouped and presented as short, standardized summaries in plain English, highlighting the study design, species involved, date, location, and main findings (Sutherland et al. 2019a). A panel of experts evaluates the certainty (strength) of the available evidence, the apparent effectiveness, and any harms (adverse impacts) in a three-round anonymized expert assessment, using a modified Delphi-technique. During this process, the experts are allowed to revise their own scores after seeing a summary of scores and comments from the rest of the panel. The scores and comments are kept anonymous throughout so that the participants are not overly influenced by any single member of the panel. The scores and categories for each conservation intervention (e.g., beneficial, likely to be beneficial, or unknown effectiveness) are then published in a searchable database and in the *What Works in Conservation* annually updated book series (Sutherland et al. 2019b).

Synopses of evidence have been published for multiple habitats and taxonomic groups, including primates (Junker et al. 2017), one of the world's most endangered taxa (Estrada et al. 2017). In this article, we quantitatively summarize the evidence published in the primate conservation synopsis and compare our results with those related to other taxonomic groups for which conservation synopses have been compiled (i.e., birds, amphibians, bees, and bats). We evaluate the evidence base in terms of the robustness of the studies that evaluated the interventions for primates, as well as the potential taxonomic, geographic, and thematic biases that may exist in the data. We describe barriers to and disincentives for testing primate conservation interventions and propose actions to improve the current evidence base for primates.

## Insufficient evidence for the effective conservation of primates

**Figure 1.**
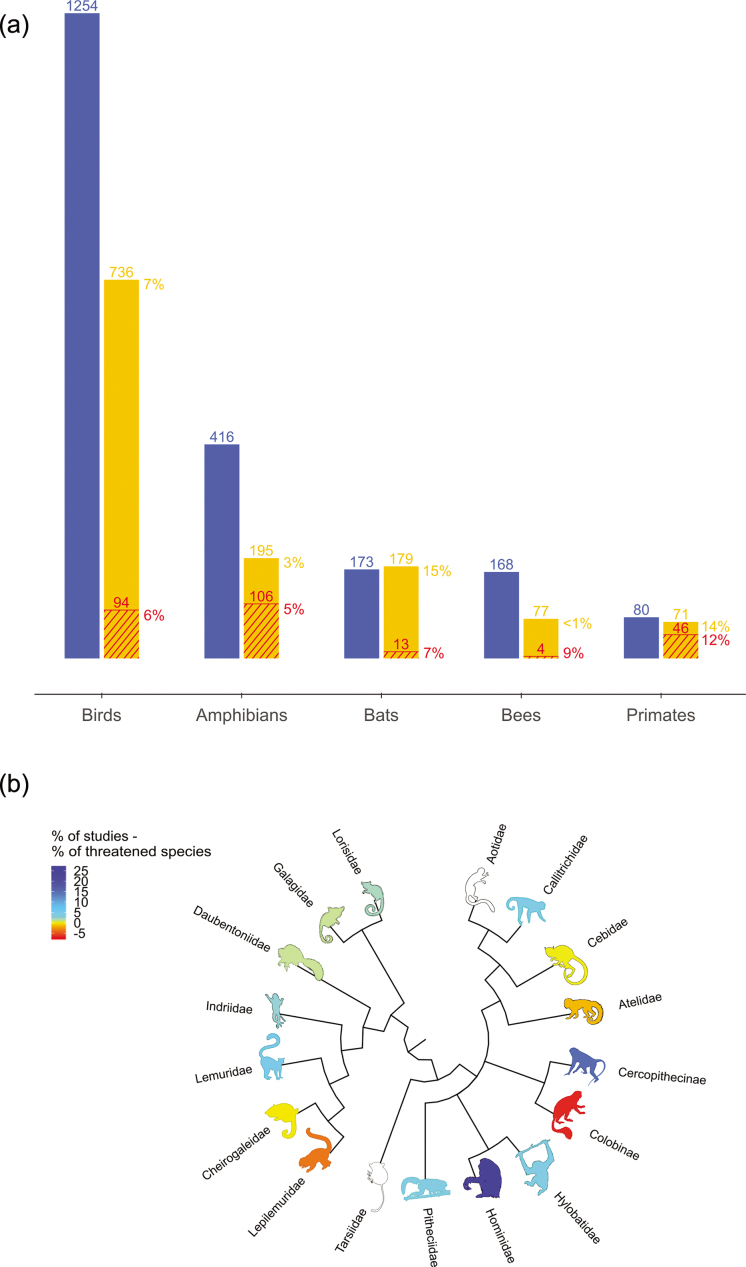
(a) Comparison of the representation of different taxa in the Conservation Evidence database showing numbers of studies evaluating conservation interventions (in blue), number of species (and percentage of total number of species) evaluated in those studies (in orange), and number of threatened (Vulnerable, Endangered or Critically Endangered on the basis of the IUCN Red List) species (and percentage of total number of threatened species) per taxonomic group (in red). (b) Relative representation of different primate families in the Conservation Evidence database. The darker blue the primate icon, the better represented the primate families are (relatively higher percentages of intervention studies compared with the percentage of threatened primate species they contain). The darker red the primate icon, the more poorly represented primate families are (relatively lower percentages of intervention studies compared with the percentage of threatened primate species). Primate families with a white primate icon indicate that they were not tested by any intervention studies. The phylogenetic tree is based on Perelman and colleagues (2011). Image silhouettes for this figure were kindly provided by Sarah Werning (https://creativecommons.org/licenses/by/3.0/). Terpsichores Indriidae (https://creativecommons.org/licenses/by-sa/3.0/), Roberto Díaz Sibaja (http://creativecommons.org/licenses/by/3.0/), Maky, Gabriella Skollar, Rebecca Lewis (https://creativecommons.org/licenses/by-sa/3.0/).

Despite the wealth of primatological literature, including well-established primate-only journals, little scientific evidence for the effectiveness of conservation of primates has been published. This is surprising, because primates (especially great apes) receive more research attention than other taxonomic groups, owing largely to their charisma and anthropological significance (Marshall et al. 2016). Of the approximately 13,000 studies published between 1971 and 2015 in 21 primate specialist journals and newsletters examined as part of the primate conservation synopsis (for details on the methodology, see Junker et al. 2017), in only 80 studies (less than 1%) was the effectiveness of primate conservation interventions investigated—very few compared with other taxa (figure 1a).

Although the proportion of threatened primate species covered by conservation intervention assessments is greater than that for other (much broader and speciose) taxa, it still amounts to only 12% of threatened primates (46 of 398 primate species classed as Vulnerable, Endangered, or Critically Endangered; IUCN 2020; figure 1a). Overall, only 14% (71 of 509) of all primate species recognized today are included, and considerable taxonomic biases are apparent; entire families are omitted from the primate conservation evidence database (e.g., Tarsiidae, Aotidae; figure 1b). Furthermore, intervention studies focused on large-bodied primates and Old World monkeys, particularly great apes (Hominidae; figure 1b). Threat status, however, did not affect study effort, although 67% of the species studied were classified as Threatened (IUCN 2020). We therefore lack the evidence-based information necessary to effectively protect and manage many vulnerable species.

**Figure 2.**
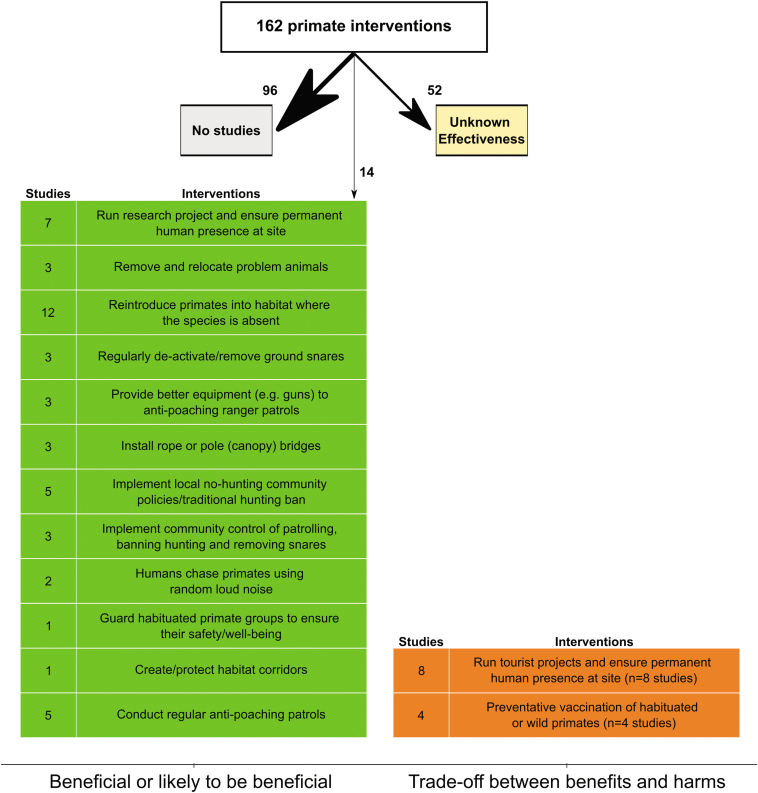
A breakdown of the number of primate interventions and corresponding studies in the Conservation Evidence database that have been assessed for their effectiveness: effective (interventions that were scored as likely to be beneficial) ineffective, or harmful (interventions that were scored as unlikely to be beneficial, that were a trade-off between benefits and harms, or that were likely to be ineffective or harmful), unknown effectiveness (studies with insufficient evidence).

We found that fewer than half (41%) of the 162 primate conservation interventions identified by primate experts in the primate conservation synopsis (Junker et al. 2017) were evaluated quantitatively (figure 2), and of those, most were assessed by hands-on practices (e.g., captive breeding and reintroductions, provisioning, habituation), which are relatively expensive and human-resource intensive and which spur much ethical debate (Fedigan 2010, Williamson and Feistner 2011, Wilson et al. 2014). People frequently assume that more effective interventions are costlier and vice versa (Neugebauer 2018), which may result in a preference for costly interventions over less expensive but potentially more effective interventions, thereby poorly prioritizing already insufficient conservation funding.

**Figure 3.**
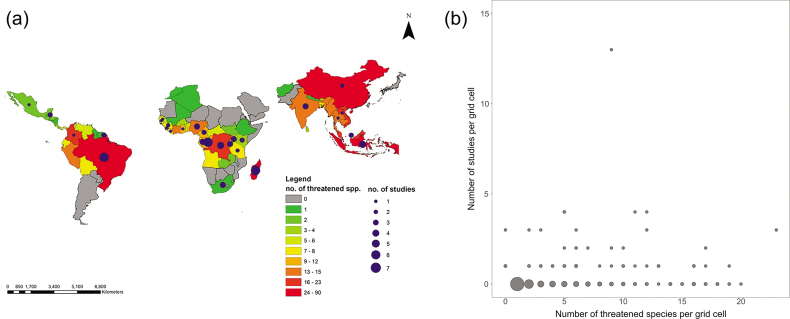
(a) The geographical distribution of the numbers of threatened primate species and of studies on primate conservation effectiveness per primate range country (data on species distribution and IUCN threat status courtesy of Anthony Rylands). The countries are color coded across their entire territories, including some areas without primates. Countries without circles had no studies reported for them. (b) The number of primate intervention studies compared with the number of threatened primate species in 2 × 2 degree grid cells. The point size represents the number of points (grid cells) at that position on the figure. Grid cells where no threatened primate species and no studies were present, were excluded.

Using evidence for 66 interventions for primates, the three-round, anonymized expert assessment (Sutherland et al. 2019a) showed that 52 (79%) were of unknown effectiveness (because of small samples, insufficient statistical testing, simultaneously implemented interventions; figure 2). This implies that many interventions are implemented without knowing whether they worked or not—an alarming result, given the urgent need for effective conservation measures. This lack of scientific evidence was also substantially higher than that for birds (56%, 210 of 374; Williams et al. 2012), bats (63%, 47 of 75; Berthinussen et al. 2014) and that for amphibians (24%, 24 of 98; Smith and Sutherland 2014). Moreover, South America and Asia were underrepresented in terms of research effort, especially when considering the number of threatened primate species living in these regions (figure 3a). We also found that the sites where many intervention studies were conducted were located in areas inhabited by relatively few threatened primate species (as has been found for threatened amphibian and bird species; Christie et al. 2020a), stressing the need for more systematic prioritization of research effort toward areas where many primate species are in need for effective conservation measures (figure 3b).

These results are alarming, given the extensive threats primates face (Estrada et al. 2017). These threats range from habitat loss due to agriculture, logging, livestock farming, mining, and infrastructure development, pollution, and climate change to hunting, trapping, and anthroponotic diseases. One might argue that conservation interventions that are effective for other taxa could be applicable to primates. However, primates have slow life histories, low reproductive rates, and high energy demands (Marshall et al. 2016), so some interventions that are effective for other species are inappropriate for primates. Primates are hunted and captured—often illegally—as pets and for medical research and are particularly vulnerable to human diseases because of our phylogenetic proximity (Estrada et al. 2017). Their arboreal habits make most primates especially vulnerable to forest loss and reduce their ability to survive in forest patches surrounded by treeless anthropogenic lands (Galán-Acedo et al. 2019). Furthermore, primate social complexity may also make them more vulnerable to population decline and extinction (Dobson and Lyles 1989, Cowlishaw et al. 2009). For example, primates that live in small family groups are more prone to demographic extinction than are more promiscuous groups, because of density-dependent effects on resource limitation (Dobson and Lyles 1989). Therefore, assessing primate conservation effectiveness on the basis of other taxa can be problematic, because primates are often specifically targeted or suffer particular risks more often than other taxa in the same habitats. Similarly, because species-specific biological traits influence how populations respond to different threats (Cowlishaw et al. 2009), the effectiveness of conservation interventions may also differ among different primate species and even within the same species in different regions or at different points in time (Christie et al. 2020b). Combined, these threats and traits suggest that primates require conservation interventions to be targeted and appropriate to their biological and social needs.

## Reasons for a lack of evidence

Although the barriers and disincentives we identify below are not unique to primates, the charismatic nature of primates and their close connection to human evolution mean there is a particularly rich literature on their behavior, phylogeny, and ecology that could be harnessed to inform targeted intervention studies. However, this literature cannot replace actual testing of interventions to understand whether interventions work or not.

### Barriers

Primate range countries are typically undergoing rapid economic development and human population growth (Estrada et al. 2018). These conditions cause habitat loss, overexploitation of resources, and increased hunting of and trade in primates (Cowlishaw and Dunbar 2000, Hansen et al. 2013). Conservation research in developing countries is often a low priority (Estrada et al. 2018), given the unmet needs of people, the lack of technical and quantitative resources in government agencies, insufficient funding, and inadequate infrastructure. A lack of collaboration between local scientists and members of the international community also reduces opportunities for local research, capacity building, and training (Sodhi and Liow 2000, Fazey et al. 2005, Mammides et al. 2016). Primates tend to occur at low densities, have slow life histories, and are difficult to count (Dobson and Lyles 1989). Population change assessments in the evaluation of conservation interventions therefore require innovative methods and intense monitoring over long periods, specific knowledge, and expertise, as well as hard to obtain long-term funding.

At many field research sites, conservation research is a by-product born of necessity, because researchers witness their study animals disappearing (e.g., Campbell et al. 2011). Primate conservationists may feel pressured to engage in several very different conservation actions simultaneously (e.g., increased ranger patrolling, translocations, captive breeding and reintroductions, habituation for tourism, community projects) because of the critical nature of the threats to the population or the short-term allocation of funding, which makes testing the effectiveness of individual interventions particularly challenging. Finally, funding agencies may bias the allocation of research funds toward specific species (Halpern et al. 2006), sites, or interventions, resulting in taxonomic, thematic, or geographic biases. This type of bias has been documented in the field of public or global health, where funding sources are often partial to more novel or technology-intensive interventions rather than those that are the most effective. For example, funding for household latrines in rural communities in Africa and Asia may be one of the more effective public health interventions, but it is not an attractive intervention for philanthropists to fund (Martinsen 2008). Similarly, an interview survey of experts on western chimpanzee (*Pan troglodytes verus*) conservation indicated that donors clearly favored interventions related to integrated conservation and development projects over projects that strengthened law enforcement, despite the lack of evidence for the effectiveness of the former (Neugebauer 2018).

### Disincentives

Publishing effectiveness evaluations for primate conservation actions can be time and resource intensive and difficult to achieve in (high impact) science journals, particularly when they show that a conservation action was not effective. Similarly, none of the interventions in the primate synopsis were assessed by the primate expert panel as unlikely to be beneficial or likely to be ineffective or harmful, and only two studies indicated that the interventions they tested were a trade-off between benefits and harms. In addition, the vast majority of the studies (46%) included in the primate synopsis were published in journals with no impact factor, and only 24% of the studies had impact factors that were greater than 2. This may discourage primatologists from pursuing a research career in primate conservation. Consequently, testing of conservation interventions for primates may lack the quality to draw clear conclusions or be buried in reports that do not undergo peer review and remain largely unknown and inaccessible.

## Toward a better assessment of primate conservation interventions

**Figure 4.**
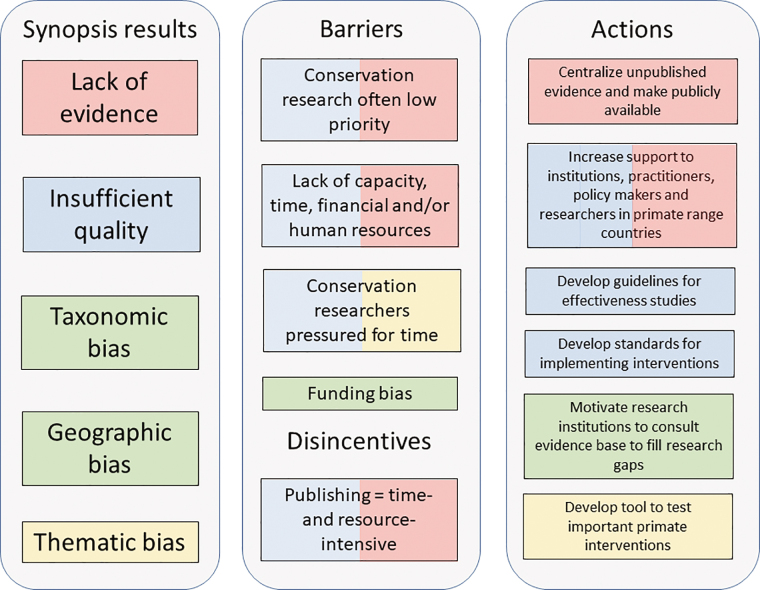
A diagram outlining the barriers and disincentives hampering evidence-based primate conservation and the actions needed to develop a more effective primate conservation framework. The colors of the different barriers and actions indicate which one of the findings of this study they relate to.

Developing a more effective primate conservation framework will be very difficult without sound knowledge of the impacts of interventions. For research to have positive impacts on conservation policy, coordination between researchers and site managers is paramount. We therefore propose specific solutions to improve the primate conservation evidence base (figure 4).

### Increase the evidence base and its use

Conservation funding bodies should target some resources specifically to intervention-effectiveness testing and publication. For example, in fiscal year 2019, the Arcus Foundation and the United States Fish and Wildlife Service awarded approximately US$18 million in grants for ape conservation and research. If these two donors agreed to a common evaluation framework to be used by grant recipients, then this could substantially improve the evidence base for apes. Moreover, nongovernmental organizations (NGOs), governments, and other relevant bodies should make more effort to evaluate conservation interventions, ideally independently, and should share the outcomes in detail (e.g., quantitative assessments of what has worked and what has not). Four of the largest conservation NGOs—Conservation International, The Nature Conservancy, the Wildlife Conservation Society, and the World Wide Fund for Nature—have already improved their efforts to generate and integrate evidence into decision-making (McKinnon et al. 2015, Dasgupta 2017). However, although the number of impact evaluations by conservation NGOs has grown, published accounts remain relatively scarce (McKinnon et al. 2015). A central location (e.g., Conservation Evidence, IUCN SSC A.P.E.S. Portal http://apesportal.eva.mpg.de) for public access to tested and reviewed—but unpublished—­primate conservation interventions could become a very valuable resource for practitioners.

Primate conservationists should consult the available evidence to prevent evidence complacency—the implementation of ineffective conservation solutions in spite of available knowledge (Sutherland and Wordley 2017). For example, orangutans (*Pongo* spp.) are rescued, rehabilitated, and translocated, and, although these strategies can improve welfare and generate income and media attention, they are expensive and provide a low return on investment and ultimately draw funding, political will, and public attention away from more effective conservation strategies, such as monitoring and enforcement, education, or protected area management (Wilson et al. 2014, Morgans et al. 2019, Sherman et al. 2020).

This is supported by the evidence compiled in the primate synopsis in that several law-enforcement interventions (e.g., regular antipoaching patrols, safeguarding habituated individuals, regular snare removal, local no-hunting policies, and traditional hunting bans, providing antipoaching ranger patrols with better equipment) were scored by the primate expert panel as likely to be beneficial (figure 2; Sutherland et al. 2019a). This is an important result, because these types of interventions are also typically less expensive than others (Neugebauer 2018, Morgans et al. 2019). Habitat protection and permanent human presence in the form of a research station on site were interventions that also received high scores for their effectiveness. In contrast to the study on orangutans by Morgans and colleagues (2019), interventions relating to education and raising awareness had no evidence or were scored as having unknown effectiveness, meaning that although evidence existed, it was inconclusive.

The Conservation Evidence project has started an initiative called the Evidence Champions. This initiative motivates companies, organizations, institutions, journals, and individuals to increase the use of conservation evidence in project planning, test interventions and publish the results, direct readers of their webpages directly to Conservation Evidence, or encourage authors to use the Conservation Evidence database when submitting articles (Sutherland et al. 2019a). An important addition to the Evidence Champions could be financial institutions that support development projects in primate habitat. Many such projects have to follow strict environmental standards to mitigate or compensate for the environmental impact caused by their activities, in order to receive financial support by international finance agencies. For example, the International Finance Corporation environmental standards include Performance Standard Six (PS6), which has recently been revised to include specific recommendations for great apes (www.primate-sg.org/PS6). Urging private companies to incorporate evaluation studies into their projects, as well as consulting and contributing to the Conservation Evidence database could be an important addition to the PS6.

Another recent development has been the linking of the IUCN Red List of Threatened Species (www.iucnredlist.org)—the world's most comprehensive information source on the global conservation status of animal, fungus and plant species—to the Conservation Evidence database. For every primate species on the IUCN Red List, the Red List now also displays both individual studies and conservation interventions, which have been compiled at Conservation Evidence.

### Fill research gaps

Future conservation effectiveness research needs added focus on threatened species, understudied regions, and conservation interventions with insufficient or no evidence (Christie et al. 2020b), especially those implemented frequently despite a lack of evidence for their effectiveness. For example, a recent study that evaluated conservation efforts for western chimpanzees showed that conservation managers’ decisions about which interventions to implement were motivated largely by their perception of the interventions’ effectiveness rather than data (Neugebauer 2018). The Conservation Evidence project in collaboration with the IUCN SSC Primate Specialist Group is planning a study to identify robust and pragmatic methods to prioritize and test important primate conservation actions that currently lack evidence. In addition, the IUCN SSC Primate Specialist Group could act as a catalyst to promote research of understudied species, regions and conservation actions. This could be done by publishing a set of research gaps for specific taxa—information that should also be included in primate conservation action plans.

### Increase the quality of intervention studies

There is a clear need for guidelines for conservation practitioners, researchers, NGOs, and the private sector for rigorous testing of primate conservation interventions and reporting their effectiveness, as well as standards of implementation. For example, interventions should ideally be tested separately to understand which intervention is working or not and whether they are sustainable across time, even if several interventions will eventually be implemented together. Similarly, it is important to use appropriate data collection methods to adequately measure effectiveness. For example, measuring the number of participants, or people's knowledge gain or attitude change are not robust measures of the effectiveness of an education or awareness campaign, because none of these measures necessarily imply human behavioral change. Moreover, simulations have shown that using simple study designs can strongly bias the results of studies, regardless of the sample size used (Christie et al. 2019).

The free PRISM Toolkit (www.conservationevaluation.org) can help practitioners design robust studies to scientifically test interventions and adequately report effectiveness results. The toolkit has been designed to help users overcome frequently encountered challenges, such as limited budgets and resources available for evaluation, short timeframes, limited technical capacity, and complex environments that can render analyses to separate project impacts from other factors difficult. Evaluation should be included in the project from the start; however, because this is seldom the case in practice, the toolkit can also be implemented in the middle or at the end of a project, and it includes step-by-step instructions for design and implementation.

Finally, it is important to follow specific standards when implementing interventions. For instance, projects that habituate primates need to follow a set of rules intended to minimize stress and disease transmission, such as maintaining a minimum distance from the animals, wearing face masks, and establishing quarantines (e.g., Macfie and Williamson 2010, Gilardi et al. 2015). A series of best practice guidelines for great ape conservation based on the best science available has been published by IUCN (www.primate-sg.org/best_practices). Best practice recommendations are incorporated into primate conservation action plans (www.primate-sg.org/action_plans). Ultimately, such standards should be informed by scientific results, or if data are not yet available, long-term field experience. In circumstances where a conservation intervention potentially threatens the health (and survival) of primates (e.g., tourism, research) and evidence for its effectiveness is not yet available, the precautionary principle must be applied.

**Figure 5.**
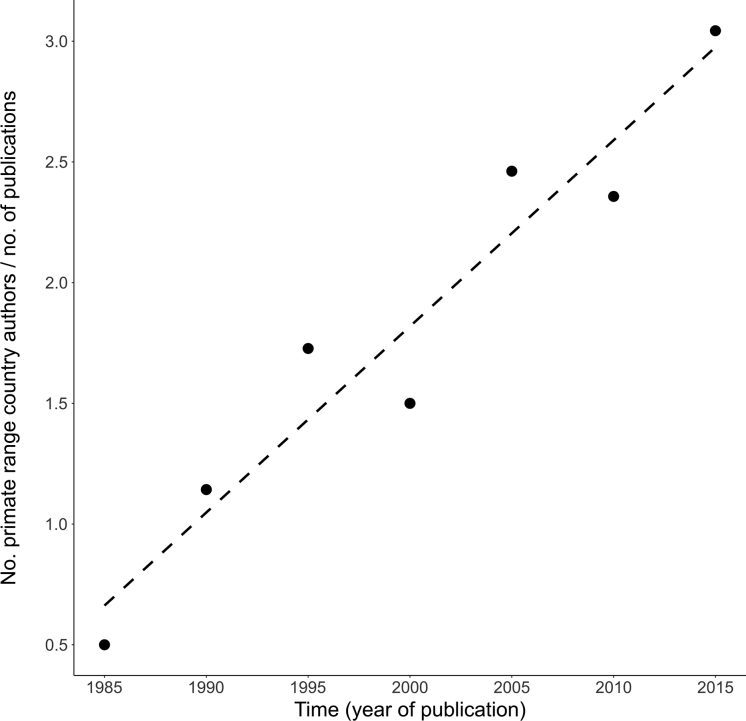
Increase in the number of primate range-country nationals authoring conservation effectiveness studies included in the primate synopsis (Junker et al. 2017) over the past 30 years (while controlling for changes in the number of publications over the same time period).

Conservation research institutions and NGOs working with primates should seek long-term collaborations and strengthen those already existing with relevant in-country institutions, field practitioners, policy makers and researchers (Christie et al. 2019). Their support should include funding conservation research infrastructure (e.g., personnel training, facilities, software and access to scientific literature). In addition, collaboration among range-country nationals should be incentivized so that more primate-focused research and conservation are led by range-country nationals to ensure long-term commitment to sites, stronger local ownership and project sustainability. It is encouraging that the number of primate range-country nationals publishing intervention studies has increased steadily over the past 30 years (figure 5), but this needs to increase substantially if we are to significantly improve the current situation. The establishment of institutions, such as the African Primatological Society (www.csrs.ch/aps/eng) is another important step in this direction. Finally, conservation research institutions should encourage their students to publish their work in scientific journals that promote applied conservation knowledge, such as *Conservation Evidence*, *Environmental Evidence*, or *Conservation Science and Practice*.

Primates are key elements of the planet's biodiversity, because of their critical ecological roles as seed dispersers and ecosystem engineers, contributing to forest regeneration and shaping the structure of plant communities, thereby changing, maintaining, or creating new habitats (Chapman et al. 2013). As the majority of primates live across the world's remaining tropical forests (Estrada et al. 2017), conserving them would also conserve a broad suite of other species in these biodiverse areas. Their phylogenetic proximity to humans plays an important role in the livelihoods, cultures, and religions of many societies and offers unique opportunities to better understand our own evolutionary history. However, we remain unable to conserve them effectively. On a positive note, the current lack of evidence offers numerous opportunities for primatologists—in collaboration with national conservation bodies and governments, international research institutions and funding bodies—to develop evidence-based strategies for conserving primates effectively. The declines of many primate species (Estrada et al. 2017) highlight the urgent need for funding and swift action to not only prevent imminent extinctions but also ensure the survival of viable primate populations in the long term. An evidence-based approach will support cost-effectiveness analysis, the prioritization of the most effective actions, and the identification of new tools to support primate conservation.

## Author Biographical

Jessica Junker (jessica.junker@idiv.de) and Hjalmar S. Kühl are affiliated with the German Centre for Integrative Biodiversity Research, in Leipzig, Germany and with the Max Planck Institute for Evolutionary Anthropology, formerly the Department of Primatology, in Leipzig, Germany. Catherine Crockford, Tene Sop, and Roman M. Wittig are affiliated with the Max Planck Institute for Evolutionary Anthropology, formerly the Department of Primatology, in Leipzig, Germany. Silviu O. Petrovan, Alec P. Christie, Rebecca K. Smith, and William J. Sutherland are affiliated with the Conservation Science Group, in the Department of Zoology at the University of Cambridge, in Cambridge, United Kingdom. Victor Arroyo-Rodríguez is affiliated with the Instituto de Investigaciones en Ecosistemas y Sustentabilidad at the Universidad Nacional Autónoma de México, in Morelia, Mexico. Ramesh Boonratana is affiliated with Mahidol University International College, in Salaya, Thailand. Dirck Byler and Russel A. Mittermeier are affiliated with Global Wildlife Conservation, in Austin, Texas. Colin A. Chapman is affiliated with the Department of Anthropology at McGill University, in Montreal, Quebec, Canada; with the School of Life Sciences at the University of KwaZulu-Natal, Scottsville, in Pietermaritzburg, South Africa; and with the Shaanxi Key Laboratory for Animal Conservation, at Northwest University, in Xi'an, China. Dilip Chetry is affiliated with the Gibbon Conservation Centre, in Assam, India. Susan M. Cheyne is affiliated with the Borneo Nature Foundation, Palangka Raya, in Central Kalimantan, Indonesia, and with the Department of Social Sciences at Oxford Brookes University, in Oxford, United Kingdom. Fanny M. Cornejo is affiliated with the Stony Brook University, in Stony Brook, New York. Liliana Cortés-Ortiz is affiliated with the Department of Ecology and Evolutionary Biology at the University of Michigan, in Ann Arbor, Michigan. Guy Cowlishaw is affiliated with the Institute of Zoology at the Zoological Society of London, in London, in the United Kingdom. Stella de la Torre is affiliated with the Universidad San Francisco de Quito's Colegio de Ciencias Biológicas y Ambientales in Quito, Ecuador. Fabiano R. de Melo is affiliated with the Department of Engenharia Florestal, at the Federal University of Viçosa, in Viçosa, Brazil. Pengfei Fan is affiliated with the School of Life Sciences at Sun Yat-Sen University, in Guangzhou, China. Cyril C. Grueter is affiliated with the School of Human Sciences and with the School of Biological Sciences at The University of Western Australia, in Crawley, Western Australia, Australia. Diana C. Guzmán-Caro is affiliated with the Asociación Primatológica Colombiana, in Bogotá, Colombia. Eckhard W. Heymann and Christian Roos are affiliated with the Deutsches Primatenzentrum at the Leibniz-Institut für Primatenforschung, in Göttingen, Germany. Ilka Herbinger is affiliated with the World Wide Fund for Wildlife Germany, in Berlin, Germany. Minh D. Hoang is affiliated with the Southern Institute of Ecology, in Hochiminh City, Vietnam. The late Robert H. Horwich was affiliated with the Community Baboon Sanctuary, Belize. Tatyana Humle is affiliated with the Durrell Institute of Conservation and Ecology, in the School of Anthropology and Conservation at the University of Kent, in Kent, in the United Kingdom. Rachel A. Ikemeh is affiliated with the SW/Niger Delta Forest Project, part of the Foundation for Sustainability of Ecosystem, Wildlife, and Climate, in Abuja, Nigeria. Inaoyom S. Imong is affiliated with the Wildlife Conservation Society Nigeria, in Calabar, Nigeria. Leandro Jerusalinsky is affiliated with the Centro Nacional de Pesquisa e Conservação de Primatas Brasileiros, in the Instituto Chico Mendes de Conservação da Biodiversidade. In João Pessoa, Brazil. Steig E. Johnson is affiliated with the Department of Anthropology and Archaeology at the University of Calgary, in Calgary, Alberta, Canada. Peter M. Kappeler is affiliated with the Deutsches Primatenzentrum at the Leibniz-Institut für Primatenforschung, in Göttingen, Germany, and with the Department of Sociobiology/Anthropology, Faculty of Biology and Psychology, at Georg-August Universität, in Göttingen, Germany. Cecilia M. Kierulff is affiliated with the Instituto Nacional da Mata Atlântica, in Espírito Santo, Brazil, and with the Instituto Pri-Matas, in Minas Gerais, Brazil. Inza Koné is affiliated with the Centre Suisse de Recherches Scientifiques, in Abidjan, Côte d'Ivoire. Rebecca Kormos is affiliated with the Department of Integrative Biology at the University of California, Berkeley. Khac Q. Le is a freelance wildlife consultant in Hanoi, Vietnam. Baoguo G. Li is affiliated with the Shaanxi Key Laboratory for Animal Conservation, in the College of Life Sciences at Northwest University, in Xi'an, China. Andrew J. Marshall is affiliated with the Department of Anthropology and the Department of Ecology and Evolutionary Biology in the Program in the Environment and the School of Environment and Sustainability, at the Universit of Michigan in Ann Arbor, Michigan. Erik Meijaard is affiliated with the Center of Excellence for Environmental Decisions, at the University of Queensland, in Brisbane, Queensland, Australia, and with Borneo Futures, in Bandar Seri Begawan, in Brunei. Yasuyuki Muroyama is affiliated with the Natural Science Laboratory, in the Faculty of Business Administration at Toyo University, in Tokyo, Japan. Eleonora Neugebauer is affiliated with the Universität Leipzig, in the Dekanat der Fakultät für Lebenswissenschaften, in Leipzig, Germany. Lisa Orth is an independent researcher in Leipzig, Germany. Erwin Palacios is affiliated with the Conservación Internacional, in Colombia, Bogotá. Sarah K. Papworth is affiliated with the Royal Holloway University of London, in Egham, in the United Kingdom. Andrew J. Plumptre is Head of the Key Biodiversity Areas Secretariat and is affilliated with BirdLife International, both in Cambridge, in the United Kingdom. Ben M. Rawson is affiliated with the World Wide Fund for Wildlife Vietnam, in Hanoi, Vietnam. Johannes Refisch is affiliated with the Great Apes Survival Partnership, in the United Nations Environment Programme, in Nairobi, Kenya. Jonah Ratsimbazafy is affiliated with the Groupe d’étude et de recherche sur les primates, in Antananarivo, Madagascar. Joanna M. Setchell is affiliated with the Department of Anthropology at Durham University, in Durham, in the United Kingdom. Christoph Schwitzer is affiliated with the Bristol Zoological Society, at the Bristol Zoo Gardens, in Bristol, in the United Kingdom. Kathy Slater is affiliated with Operation Wallacea, in Lincolnshire, in the United Kingdom. Shirley C. Strum is affiliated with the University of California San Diego, in La Jolla, California, and with the Uaso Ngiro Baboon Project, in Nairobi, Kenya. Maurício Talebi is affiliated with the Departamento de Cíências Ambientais and the Programa Análise Ambiental Integrada, at the Universidade Federal de São Paulo, in Sao Paulo, Brazil. Janette Wallis is affiliated with the Department of Environmental Studies at the University of Oklahoma, in Norman, Oklahoma. Serge Wich is affiliated with the School of Natural Sciences and Psychology at Liverpool John Moores University, in Liverpool, in the United Kingdom. Elizabeth A. Williamson is affiliated with the Faculty of Natural Sciences at the University of Stirling, in Stirling, Scotland, in the United Kingdom. Roman M. Wittig are and Catherine Crockford are also affiliated with the Tai Chimpanzee Project, at the Centre Suisse des Recherche Scientifique, in Abidjan, Cote d'Ivoire.
