# Corrigendum: A Severe Lack of Evidence Limits Effective Conservation of the World's Primates

**DOI:** 10.1093/biosci/biaa143

**Published:** 2020-11-13

**Authors:** Jessica Junker, Silviu O Petrovan, Victor Arroyo-Rodríguez, Ramesh Boonratana, Dirck Byler, Colin A Chapman, Dilip Chetry, Susan M Cheyne, Fanny M Cornejo, Liliana Cortés-Ortiz, Guy Cowlishaw, Alec P Christie, Catherine Crockford, Stella de la Torre, Fabiano R de MELO, P Fan, Cyril C Grueter, Diana C Guzmán-Caro, Eckhard W Heymann, Ilka Herbinger, Minh D Hoang, Robert H Horwich, Tatyana Humle, Rachel A Ikemeh, Inaoyom S Imong, Leandro Jerusalinsky, Steig E Johnson, Peter M Kappeler, Maria Cecília M Kierulff, Inza Koné, Rebecca Kormos, Khac Q LE, Baoguo Li, Andrew J Marshall, Erik Meijaard, Russel A Mittermeier, Yasuyuki Muroyama, Eleonora Neugebauer, Lisa Orth, Erwin Palacios, Sarah K Papworth, Andrew J Plumptre, Ben M Rawson, Johannes Refisch, Jonah Ratsimbazafy, Christian Roos, Joanna M Setchell, Rebecca K Smith, Tene Sop, Christoph Schwitzer, Kathy Slater, Shirley C Strum, William J Sutherland, Maurício Talebi, Janette Wallis, Serge Wich, Elizabeth A Williamson, Roman M WITTIG, Hjalmar S Kühl

**Affiliations:** Department of Primatology, Leipzig, Germany; Department of Zoology, University of Cambridge, Cambridge, United Kingdom; Universidad Nacional Autónoma de México, Morelia, Mexico; Mahidol University International College, Salaya, Thailand; Global Wildlife Conservation, Austin, Texas; Department of Anthropology, McGill University, Montreal, Quebec, Canada; with the School of Life Sciences, University of KwaZulu-Natal, Scottsville, Pietermaritzburg, South Africa; and with the Shaanxi Key Laboratory for Animal Conservation, Northwest University, in Xi'an, China; Gibbon Conservation Centre, Assam, India; Department of Social Sciences, Oxford Brookes University, Oxford, United Kingdom; Stony Brook University, Stony Brook, New York; Department of Ecology and Evolutionary Biology, University of Michigan, Ann Arbor, Michigan; Zoological Society of London, London, United Kingdom; Department of Zoology, University of Cambridge, Cambridge, United Kingdom; Department of Primatology, Leipzig, Germany; Tai Chimpanzee Project, Centre Suisse des Recherche Scientifique, Abidjan, Cote d'Ivoire; Universidad San Francisco de Quito's Colegio de Ciencias Biológicas y Ambientales Quito, Ecuador; Department of Engenharia Florestal, Federal University of Viçosa, Viçosa, Brazil; Sun Yat-Sen University, Guangzhou, China; University of Western Australia, Crawley, Western Australia, Australia; Asociación Primatológica Colombiana, Bogotá, Colombia; Deutsches Primatenzentrum, Leibniz-Institut für Primatenforschung, Göttingen, Germany; World Wide Fund for Wildlife Germany, Berlin, Germany; Southern Institute of Ecology, Hochiminh City, Vietnam; Community Baboon Sanctuary, Belize; Durrell Institute of Conservation and Ecology, School of Anthropology and Conservation, University of Kent, in Kent, United Kingdom; SW/Niger Delta Forest Project, part of the Foundation for Sustainability of Ecosystem, Wildlife, and Climate, Abuja, Nigeria; Wildlife Conservation Society Nigeria, Calabar, Nigeria; Centro Nacional de Pesquisa e Conservação de Primatas Brasileiros, Instituto Chico Mendes de Conservação da Biodiversidade. In João Pessoa, Brazil; Department of Anthropology and Archaeology, University of Calgary, Calgary, Alberta, Canada; Department of Sociobiology/Anthropology, Faculty of Biology and Psychology, Georg-August Universität, Göttingen, Germany; Instituto Nacional da Mata Atlântica, Espírito Santo, Brazil, and with the Instituto Pri-Matas, in Minas Gerais, Brazil; Department of Integrative Biology, University of California, Berkeley; Department of Primatology, Leipzig, Germany; Freelance wildlife consultant Hanoi, Vietnam; Shaanxi Key Laboratory for Animal Conservation, College of Life Sciences, Northwest University, Xi'an, China; Department of Anthropology, Department of Ecology and Evolutionary Biology in the Program in the Environment and the School of Environment and Sustainability, Universit of Michigan, Ann Arbor, Michigan; University of Queensland, Brisbane, Queensland, Australia, and with Borneo Futures, in Bandar Seri Begawan, Brunei; Global Wildlife Conservation, Austin, Texas; Natural Science Laboratory, Faculty of Business Administration, Toyo University, in Tokyo, Japan; Universität Leipzig, Dekanat der Fakultät für Lebenswissenschaften, Leipzig, Germany; Independent researcher Leipzig, Germany; Conservación Internacional, Colombia, Bogotá; Royal Holloway University of London, Egham, United Kingdom; BirdLife International, Cambridge, United Kingdom; World Wide Fund for Wildlife Vietnam, Hanoi, Vietnam; Great Apes Survival Partnership, United Nations Environment Programme, Nairobi, Kenya; Groupe d’étude et de recherche sur les primates, Antananarivo, Madagascar; Deutsches Primatenzentrum, Leibniz-Institut für Primatenforschung, Göttingen, Germany; Department of Anthropology, Durham University, Durham, United Kingdom; Department of Zoology, University of Cambridge, Cambridge, United Kingdom; Department of Primatology, Leipzig, Germany; Bristol Zoological Society, Bristol Zoo Gardens, Bristol, United Kingdom; Operation Wallacea, Lincolnshire, United Kingdom; University of California San Diego, La Jolla, California, and with the Uaso Ngiro Baboon Project, Nairobi, Kenya; Department of Zoology, University of Cambridge, Cambridge, United Kingdom; Departamento de Cíências Ambientais, Programa Análise Ambiental Integrada, Universidade Federal de São Paulo, Sao Paulo, Brazil; Department of Environmental Studies, University of Oklahoma, Norman, Oklahoma; School of Natural Sciences and Psychology, Liverpool John Moores University, Liverpool, United Kingdom; Faculty of Natural Sciences, University of Stirling, Stirling, Scotland, United Kingdom; Department of Primatology, Leipzig, Germany; Tai Chimpanzee Project, Centre Suisse des Recherche Scientifique, Abidjan, Cote d'Ivoire; Department of Primatology, Leipzig, Germany

In the originally published version of this article, the author's name, Kathy Slater, was incorrectly spelt in the author list and within the “Author Biographical” section.  This has now been corrected online.

## Author Biographical

Jessica Junker (jessica.junker@idiv.de) and Hjalmar S. Kühl are affiliated with the German Centre for Integrative Biodiversity Research, in Leipzig, Germany and with the Max Planck Institute for Evolutionary Anthropology, formerly the Department of Primatology, in Leipzig, Germany. Catherine Crockford, Tene Sop, and Roman M. Wittig are affiliated with the Max Planck Institute for Evolutionary Anthropology, formerly the Department of Primatology, in Leipzig, Germany. Silviu O. Petrovan, Alec P. Christie, Rebecca K. Smith, and William J. Sutherland are affiliated with the Conservation Science Group, in the Department of Zoology at the University of Cambridge, in Cambridge, United Kingdom. Victor Arroyo-Rodríguez is affiliated with the Instituto de Investigaciones en Ecosistemas y Sustentabilidad at the Universidad Nacional Autónoma de México, in Morelia, Mexico. Ramesh Boonratana is affiliated with Mahidol University International College, in Salaya, Thailand. Dirck Byler and Russel A. Mittermeier are affiliated with Global Wildlife Conservation, in Austin, Texas. Colin A. Chapman is affiliated with the Department of Anthropology at McGill University, in Montreal, Quebec, Canada; with the School of Life Sciences at the University of KwaZulu-Natal, Scottsville, in Pietermaritzburg, South Africa; and with the Shaanxi Key Laboratory for Animal Conservation, at Northwest University, in Xi'an, China. Dilip Chetry is affiliated with the Gibbon Conservation Centre, in Assam, India. Susan M. Cheyne is affiliated with the Borneo Nature Foundation, Palangka Raya, in Central Kalimantan, Indonesia, and with the Department of Social Sciences at Oxford Brookes University, in Oxford, United Kingdom. Fanny M. Cornejo is affiliated with the Stony Brook University, in Stony Brook, New York. Liliana Cortés-Ortiz is affiliated with the Department of Ecology and Evolutionary Biology at the University of Michigan, in Ann Arbor, Michigan. Guy Cowlishaw is affiliated with the Institute of Zoology at the Zoological Society of London, in London, in the United Kingdom. Stella de la Torre is affiliated with the Universidad San Francisco de Quito's Colegio de Ciencias Biológicas y Ambientales in Quito, Ecuador. Fabiano R. de Melo is affiliated with the Department of Engenharia Florestal, at the Federal University of Viçosa, in Viçosa, Brazil. Pengfei Fan is affiliated with the School of Life Sciences at Sun Yat-Sen University, in Guangzhou, China. Cyril C. Grueter is affiliated with the School of Human Sciences and with the School of Biological Sciences at The University of Western Australia, in Crawley, Western Australia, Australia. Diana C. Guzmán-Caro is affiliated with the Asociación Primatológica Colombiana, in Bogotá, Colombia. Eckhard W. Heymann and Christian Roos are affiliated with the Deutsches Primatenzentrum at the Leibniz-Institut für Primatenforschung, in Göttingen, Germany. Ilka Herbinger is affiliated with the World Wide Fund for Wildlife Germany, in Berlin, Germany. Minh D. Hoang is affiliated with the Southern Institute of Ecology, in Hochiminh City, Vietnam. The late Robert H. Horwich was affiliated with the Community Baboon Sanctuary, Belize. Tatyana Humle is affiliated with the Durrell Institute of Conservation and Ecology, in the School of Anthropology and Conservation at the University of Kent, in Kent, in the United Kingdom. Rachel A. Ikemeh is affiliated with the SW/Niger Delta Forest Project, part of the Foundation for Sustainability of Ecosystem, Wildlife, and Climate, in Abuja, Nigeria. Inaoyom S. Imong is affiliated with the Wildlife Conservation Society Nigeria, in Calabar, Nigeria. Leandro Jerusalinsky is affiliated with the Centro Nacional de Pesquisa e Conservação de Primatas Brasileiros, in the Instituto Chico Mendes de Conservação da Biodiversidade. In João Pessoa, Brazil. Steig E. Johnson is affiliated with the Department of Anthropology and Archaeology at the University of Calgary, in Calgary, Alberta, Canada. Peter M. Kappeler is affiliated with the Deutsches Primatenzentrum at the Leibniz-Institut für Primatenforschung, in Göttingen, Germany, and with the Department of Sociobiology/Anthropology, Faculty of Biology and Psychology, at Georg-August Universität, in Göttingen, Germany. Cecilia M. Kierulff is affiliated with the Instituto Nacional da Mata Atlântica, in Espírito Santo, Brazil, and with the Instituto Pri-Matas, in Minas Gerais, Brazil. Inza Koné is affiliated with the Centre Suisse de Recherches Scientifiques, in Abidjan, Côte d'Ivoire. Rebecca Kormos is affiliated with the Department of Integrative Biology at the University of California, Berkeley. Khac Q. Le is a freelance wildlife consultant in Hanoi, Vietnam. Baoguo G. Li is affiliated with the Shaanxi Key Laboratory for Animal Conservation, in the College of Life Sciences at Northwest University, in Xi'an, China. Andrew J. Marshall is affiliated with the Department of Anthropology and the Department of Ecology and Evolutionary Biology in the Program in the Environment and the School of Environment and Sustainability, at the Universit of Michigan in Ann Arbor, Michigan. Erik Meijaard is affiliated with the Center of Excellence for Environmental Decisions, at the University of Queensland, in Brisbane, Queensland, Australia, and with Borneo Futures, in Bandar Seri Begawan, in Brunei. Yasuyuki Muroyama is affiliated with the Natural Science Laboratory, in the Faculty of Business Administration at Toyo University, in Tokyo, Japan. Eleonora Neugebauer is affiliated with the Universität Leipzig, in the Dekanat der Fakultät für Lebenswissenschaften, in Leipzig, Germany. Lisa Orth is an independent researcher in Leipzig, Germany. Erwin Palacios is affiliated with the Conservación Internacional, in Colombia, Bogotá. Sarah K. Papworth is affiliated with the Royal Holloway University of London, in Egham, in the United Kingdom. Andrew J. Plumptre is Head of the Key Biodiversity Areas Secretariat and is affilliated with BirdLife International, both in Cambridge, in the United Kingdom. Ben M. Rawson is affiliated with the World Wide Fund for Wildlife Vietnam, in Hanoi, Vietnam. Johannes Refisch is affiliated with the Great Apes Survival Partnership, in the United Nations Environment Programme, in Nairobi, Kenya. Jonah Ratsimbazafy is affiliated with the Groupe d’étude et de recherche sur les primates, in Antananarivo, Madagascar. Joanna M. Setchell is affiliated with the Department of Anthropology at Durham University, in Durham, in the United Kingdom. Christoph Schwitzer is affiliated with the Bristol Zoological Society, at the Bristol Zoo Gardens, in Bristol, in the United Kingdom. Kathy Slater is affiliated with Operation Wallacea, in Lincolnshire, in the United Kingdom. Shirley C. Strum is affiliated with the University of California San Diego, in La Jolla, California, and with the Uaso Ngiro Baboon Project, in Nairobi, Kenya. Maurício Talebi is affiliated with the Departamento de Cíências Ambientais and the Programa Análise Ambiental Integrada, at the Universidade Federal de São Paulo, in Sao Paulo, Brazil. Janette Wallis is affiliated with the Department of Environmental Studies at the University of Oklahoma, in Norman, Oklahoma. Serge Wich is affiliated with the School of Natural Sciences and Psychology at Liverpool John Moores University, in Liverpool, in the United Kingdom. Elizabeth A. Williamson is affiliated with the Faculty of Natural Sciences at the University of Stirling, in Stirling, Scotland, in the United Kingdom. Roman M. Wittig are and Catherine Crockford are also affiliated with the Tai Chimpanzee Project, at the Centre Suisse des Recherche Scientifique, in Abidjan, Cote d'Ivoire.

